# Acoustic Cavitation Emissions Predict Near-complete/complete Histotripsy Treatment in Soft Tissues

**DOI:** 10.1016/j.ultrasmedbio.2025.02.005

**Published:** 2025-02-26

**Authors:** Scott C. Haskell, Ellen Yeats, Jiaqi Shi, Tim Hall, J. Brian Fowlkes, Zhen Xu, Jonathan R. Sukovich

**Affiliations:** a Department of Biomedical Engineering, University of Michigan, Ann Arbor, MI, USA; b Department of Pathology, University of Michigan, Ann Arbor, MI, USA; c Department of Radiology, University of Michigan, Ann Arbor, MI, USA; d Department of Neurosurgery, University of Michigan, Ann Arbor, MI, USA

**Keywords:** histotripsy, treatment monitoring, therapeutic ultrasound, cavitation

## Abstract

**Objective:**

Histotripsy is a non-invasive acoustic ablation technique that leverages cavitation to impart mechanical damage to a viscoelastic medium, such as tissue. Although histotripsy bubbles and lesions can be imaged with a variety of modalities, reliable methods to predict tissue disruption across different tissue-types remain to be determined.

**Approach:**

Several ex-vivo bovine tissues were ablated by intrinsic threshold histotripsy over a range of pulse-per-location acoustic doses. Acoustic Cavitation Emission (ACE) signals were captured following every other therapeutic pulse using transmit-receive capable histotripsy arrays. Final bubble lifespan, lifespan-slope, and percent-reduction were calculated and correlated against histologic necrosis score (0–5: 0=0% necrosis, 5=>95% necrosis) and residual structure score (0–4: 0=none present, 4=intact) to evaluate the ability of features from ACE-signals to predict histotripsy-induced damage. Further, optimal ACE-feature thresholds were determined for binary evaluation of whether a necrosis score equal or greater than 4 had been reached.

**Results:**

Measured lifespans increased and lifespan-slopes decreased with pulses per location (ppl) and eventually plateaued in all tissue types, in similar trends to those previously observed in tissue phantoms. Necrosis score increased and residual structure decreased with increasing acoustic dose. Bubble lifespan-slope and percent-reduction correlated well with necrosis score. Thresholds able to predict the necrosis score of 4 or greater in brain, liver, and kidney were calculated with high sensitivity/specificity (>80%). The necrosis score of 4 and 5 is expected to correspond to near-complete/complete ablation by histological evaluation.

**Conclusion:**

Features measured from ACE-signals, particularly the lifespan-slope and percent reduction, were used to predict near-complete/complete ablation of large-volume histotripsy treatments in ex vivo bovine liver, kidney, and brain tissues with good accuracy. Tissue heterogeneities were observed to impact the histotripsy damage and corresponding ACE-signals, and thus the predication accuracy.

## Introduction

Histotripsy is a non-invasive acoustic ablation technique that utilizes cavitation to mechanically disrupt tissues. Cavitation during histotripsy is generated using microsecond length, high-amplitude pressure pulses to excite native dissolved gas nuclei. The rapid growth and collapse of the excited gas nuclei result in high stresses and strains in tissues, which fractionates and ultimately reduces them to liquefied acellular homogenates. Histotripsy ablation of tissues has been demonstrated in a wide range of tissue types (e.g., kidney, liver, brain, blood clots) and in a wide range of locations in the body [1–10]. The first histotripsy device has recently been approved by the FDA for use in treating primary and metastatic liver tumors [11].

Although histotripsy treatments have been successfully and safely applied to liver tumors in human patients, a key challenge has been that of actively monitoring the level of induced damage (e.g., percent tumor kill) generated in tissue during histotripsy treatments, which is critical for ensuring reliable, repeatable, and efficient treatments between patients. While numerous methods exist for monitoring the generation/presence of cavitation and the area associated damage in tissues (e.g. diagnostic B-mode ultrasound imaging, MRI, passive cavitation imaging (PCI)), these methods do not confer reliable information on the intensity of the damage to the exposed media [12–16]. Histotripsy ablation zones can be visualized on B-mode ultrasound imaging as a hypoechoic zone; however, the reduced echogenicity can be offset by the coagulation of the homogenized tissue debris and cannot be used to reliably determine whether the target zone is partially or completely damaged. Additionally, MR imaging is highly costly, clinically burdensome, and is not fast enough for use in real-time during histotripsy. Alternatively, more recent work is ongoing to leverage alternative ultrasound imaging methods such as color flow imaging or elastography to localize bubble activity and possibly predict the level of induced damage [17–21].

Further complicating matters, the susceptibility of different tissues to histotripsy-induced damage is known to be highly dependent on the tissues’ mechanical properties, which can be difficult to predict a priori and varies between patients and tumor types [22,23]. In practice, the lack of reliable indicators of complete treatment (i.e. tissue homogenization), and the known variability in tissues’ susceptibility to histotripsy-induced damage, effectively necessitate the use of a worst-case-scenario treatment plan [11]. In other words: using the set of exposure conditions with sufficiently high dose determined to lead to the complete ablation of the most-difficult-to-treat tissue type in all tumors and patients. While such an approach may be effective for ensuring complete destruction of targeted tumors in all patients, it can lead to long treatment durations, particularly when targets are larger than 5 centimeters in diameter [24]. In addition, recent studies in in-vivo rodent tumor models have suggested that there is a dose-dependent, anti-tumor immune response following histotripsy treatment [25,26]. These results further highlight the need for establishing metrics and methods for monitoring histotripsy-induced damage in tissues.

The nucleation and collapse of inertial cavitation bubbles, such as those generated during histotripsy, are known to emit acoustic shock-waves during said nucleation and collapse phases. The dynamics of cavitation bubbles, including the relative timings and amplitudes of these shockwaves, are known to be dependent on the viscoelastic properties of the nucleation medium [16,27–31]. Repeated exposure to cavitation during histotripsy (i.e., increasing cavitation dose) is known to incrementally reduce tissues to liquefied acellular homogenate; by inspection, the reduction of tissue from a viscoelastic solid to a liquid homogenate will alter its viscoelastic properties. Under otherwise equivalent nucleation conditions, the dynamics of bubbles generated in the exposed tissues, and corresponding shockwaves emitted therefrom, can be expected to demonstrate measurable changes. In a previous study in which histotripsy was applied to single focal-site targets within viscoelastic hydrogels, it was demonstrated via high-speed imaging that the dynamics of the generated cavitation varied as a function of increasing levels of induced damage generated in the hydrogels, which in turn was a function of increasing acoustic exposure [32]. In particular, the bubble lifespan (time between cavitation nucleation and collapse) was observed to increase with increasing damage levels before plateauing when damage was complete. That study also demonstrated that the changes in cavitation lifespan could be detected using a transmit-receive capable histotripsy array by monitoring the Acoustically-nucleated Inertial Cavitation Emitted Shockwave signals, hereafter referred to as Acoustic Cavitation Emission (ACE) shockwave signals for brevity. Hydrophone measurements of the ACE-signals during a preliminary study in which histotripsy treatments were applied to single focal site targets in ex-vivo bovine liver tissues similarly showed that the bubble lifespan increased as a function of increasing acoustic dose before eventually plateauing beyond certain exposure thresholds [32,33], However, no post-treatment histological analyses were performed to evaluate the level of cellular destruction in these targets to quantitatively correlate them with the measured ACE-signals.

The goal of this paper was to evaluate the feasibility and accuracy of using features from ACE-signals, specifically the bubble lifespan, to predict near-complete/complete tissue disruption of large-volume, intrinsic-threshold histotripsy ablations by two ultrasound arrays (different F#’s) across several representative soft tissue types (brain, liver, kidney). ACE-signals were captured during every-other pulse of each treatment for post-hoc analysis. Following exposure, each sample was fixed, stained, and analyzed by a board-certified pathologist for the degree of tissue disruption. Pathologist scores were compared against bubble dynamics measured from the ACE-signals to measure their predictive value.

Ex-vivo bovine brain, liver, and kidney tissues were selected because their material properties are representative of the range of soft tissue histotripsy targets [23]. Although ACE-signal monitoring methods could still be developed for non-soft tissue histotripsy targets (e.g. tendon, bone), the significant difference in material properties and structure would likely generate different patterns of bubble dynamics. Another key factor in this study is that the ablations will be “large-volume” relative to the bubble cloud size (~280:1). Previous studies using ACE-signal based monitoring methods were for single-point targets, but it was unclear whether/how anisotropic cavitation conditions could affect the pattern of bubble dynamics during treatment (e.g. tissue heterogeneity like a blood vessel, damage to adjacent locations). Finally, treatments for these tissues will be performed with different two histotripsy arrays; arrays with geometries relevant to treating the respective tissues (abdominal and cranial). Array focusing is known to affect bubble cloud dynamics [34], so demonstrating whether ACE-signal monitoring methods work across these two arrays could provide some evidence of its robustness for translation to any future clinical devices.

## Materials and Methods

### Experimental setup

**Figure 1.**
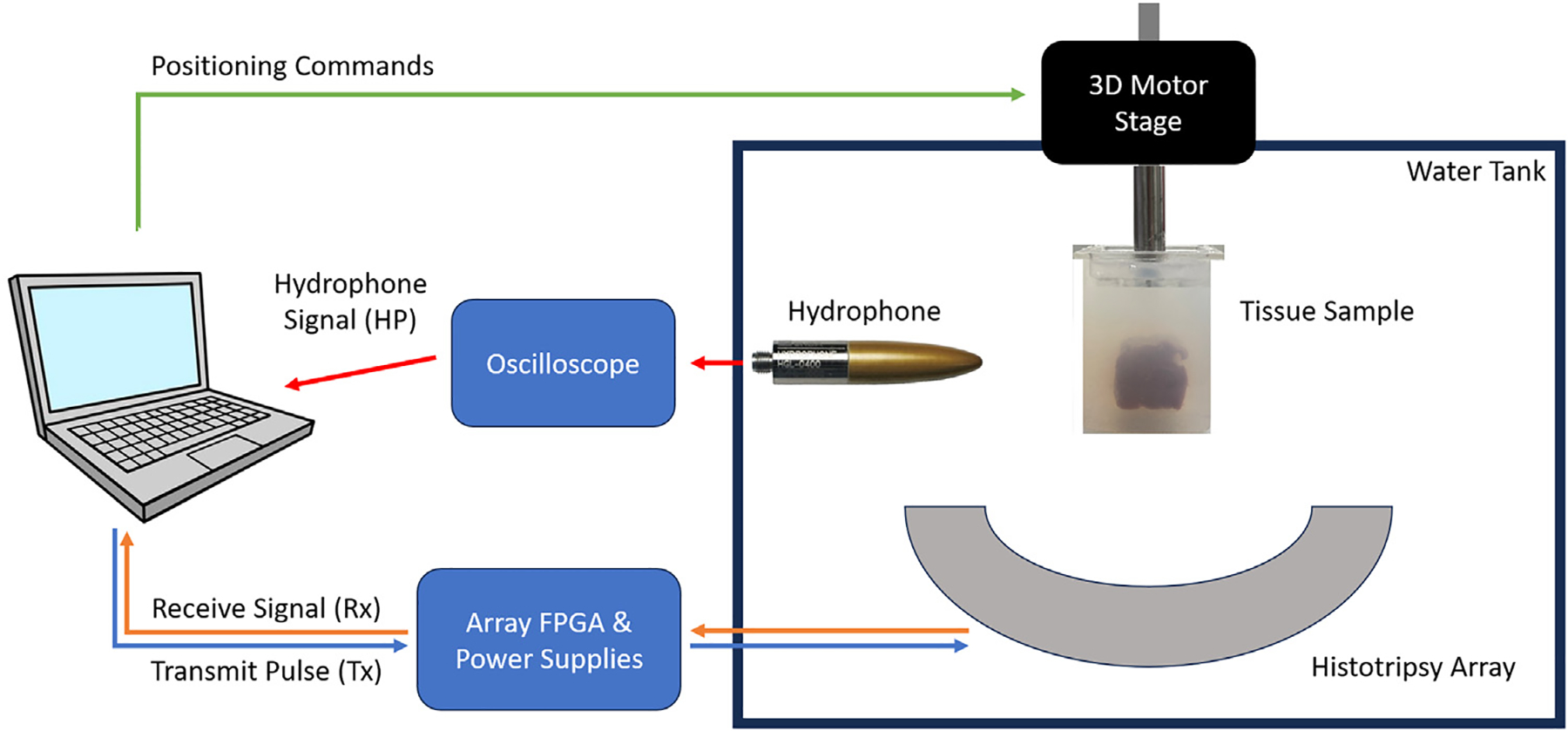
Experimental Setup Diagram.

All experiments were performed in tanks of de-ionized water filtered to 2μm and degassed to 20% oxygen saturation as measured by a ThermoFisher Orion Star A323 oxygen meter (Waltham, MA). The water temperatures were measured to be between 20 and 21°C. Samples of ex-vivo bovine tissue (liver, kidney, brain; < 2hrs from sacrifice) were procured from a local abattoir. Tissues were cut into 3 cm cubic volumes and degassed under 30inHg vacuum for at least 3 hours to extract any gas that dissolved into the tissues during transit [22]. Samples were then embedded in 1.5% agarose hydrogel tissue holders [3]. During treatments (described below), the tissue samples were mounted at the geometric focus of the histotripsy transducer in the water tank and treated using a range of different acoustic doses to generate different levels of induced damage at the treatments’ endpoints (Figure 1). All tissues were treated within four days of acquisition.

### Histotripsy transducers

**Figure 2.**
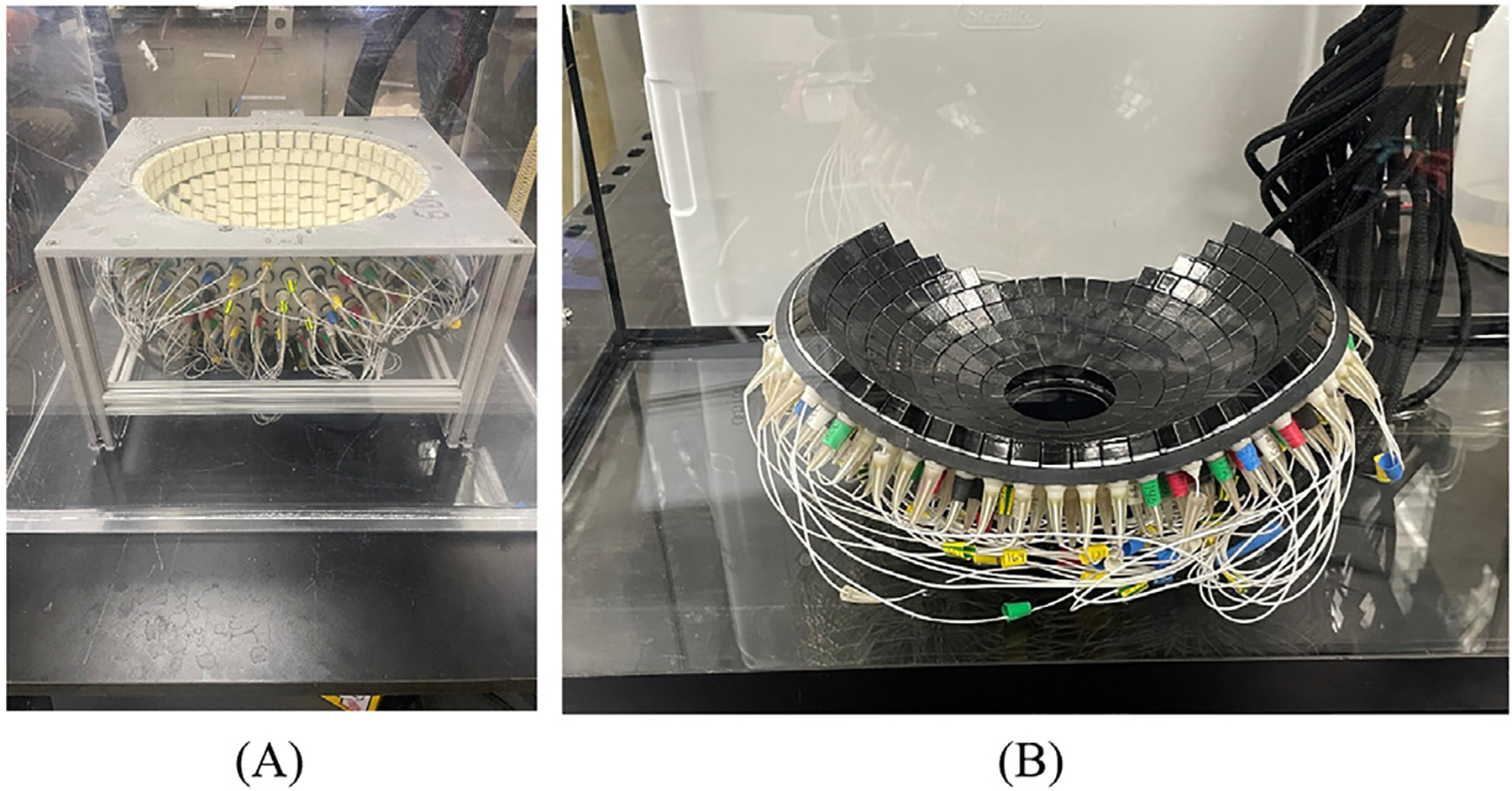
(A) 700kHz, 360-element transcranial histotripsy array used in the bovine brain experiments (15cm diameter; 17 × 17 mm^2^ elements); (B) 750kHz, 260-element abdominal histotripsy array used in the bovine liver and kidney experiments (14.2cm radius of curvature; 11.5 × 11.5 mm^2^ elements).

Two histotripsy transducers, designed and fabricated in-house, were used in this study. The transcranial array is a 360 element, 30 cm aperture diameter hemispherical histotripsy array with a center frequency of 700kHz (Figure 2A). The focal zone was 1.2 × 1.2 × 2.3mm^3^ in the two transverse and axial axes, respectively. Its geometry was designed to cater to transcranial treatments and was used to treat the bovine brain samples [2]. The abdominal histotripsy array is a spherically curved 750kHz phased array with a 14.2cm focal radius (Figure 2B). The 260 elements of the array are arranged in concentric rings around a 6 cm diameter cutout at the center to allow for the placement of an imaging transducer. The focal zone of the array was measured to be 1.6 × 1.1 × 4.5 mm^3^. The array has an ellipsoidal aperture suitable for coupling to the abdomen, so it was used to treat the bovine liver and kidney samples [35].

Each transducer element of both arrays was configured to operate in a transmit-receive mode to allow simultaneous generation of the acoustic pulses necessary to generate cavitation and the acquisition of the resulting ACE-signals emitted therefrom. The individual transducer elements were characterized by measuring their output waveform using a fiber-optic hydrophone (HP; Onda HPO-690, Sunnyvale CA) positioned at the focal radius of each array (transcranial − 15cm, abdominal 14.2cm). Acoustic pulses emitted by the elements of each array measured approximately 1.5 acoustic cycles, with corresponding durations of 2.1us (transcranial array) and 2us (abdominal array). The focal zones of the arrays were calculated by firing all of the elements and measuring one-dimensional (1D) transmit beam profiles along three axes using a needle hydrophone (Onda HNR-500, Sunnyvale CA), at low driving pressures (≤1MPa) [35,36]. The sizes of the arrays’ focal zones were defined as the full-width half-maximum (FWHM) of the measured 1D beam profiles. On receive, each transducer element of the arrays was connected to a 12-bit analog-to-digital converter (ADC) with a maximum sampling frequency of up to 50 MHz. Control of the arrays was accomplished using custom programmed system-on-a-chip (SoC) devices, which could store received ACE-signals from every element of the array after every delivered acoustic pulse.

### Histotripsy treatment parameters

An 8 × 8 × 8 mm^3^ cubic ablation region was prescribed to each tissue sample. This volume was comprised of many individual histotripsy steering foci that were arranged in a hexagonal-close-packing (HCP) lattice with 0.5 mm lattice point spacing. During treatment, the focal pressure amplitudes of the array were set to 30 and 40 MPa for the abdominal and transcranial array treatments, respectively. The different absolute pressure amplitudes between arrays were chosen to compensate for the differences in the focal zone shapes and set such that the histotripsy bubble clouds generated by each array would be roughly equivalent in size and have diameters of ~1.5mm. These pressures were determined from preliminary studies in which brain, liver, and kidney samples were exposed to up to 50 pulses of histotripsy at a single focal location at varying driving pressures, processed for H&E histology, and analyzed to compare the relationship how driving pressure determines focal lesion size for each array. Further, the electric drive power of the arrays was modulated during treatment to compensate for focal steering pressure losses such that these pressures were uniformly maintained throughout the entire steering volume. During treatments, histotripsy pulses were applied to the tissue samples at a pulse repetition frequency (PRF) of 500 Hz. Treatments were delivered such that each subsequent histotripsy pulse would be applied to a different focal location within the steering grid until one pulse had been delivered to all steering locations within the grid. This was then repeated a specified number of times to meet desired acoustic dose, hereafter referred to as pulses-per-location (ppl), of the treatment. In each tissue type, exposures of 3 / 5 / 10 / 20 / 30 ppl were delivered; 4 brain samples and 6 liver and kidney samples were treated per exposure level (80 tissue samples total). To ensure that the cut plane of the tissue during histological sectioning bisected the generated ablation zone, an additional ‘registration’ lesion was generated by applying 500 histotripsy pulses along the line between [0,0,−15] and [0,0,−30] (i.e. the boundary between gel & tissue). These ‘registration’ lesions were placed ≥10mm from the cubic treatment volume, measured approximately 2mm in diameter, were aligned with the center of the cubic treatment volume along its transverse (X, Y) axes, and were visible to the naked eye to assist with histological sectioning.

### Acoustic cavitation emission (ACE) signals

**Figure 3.**
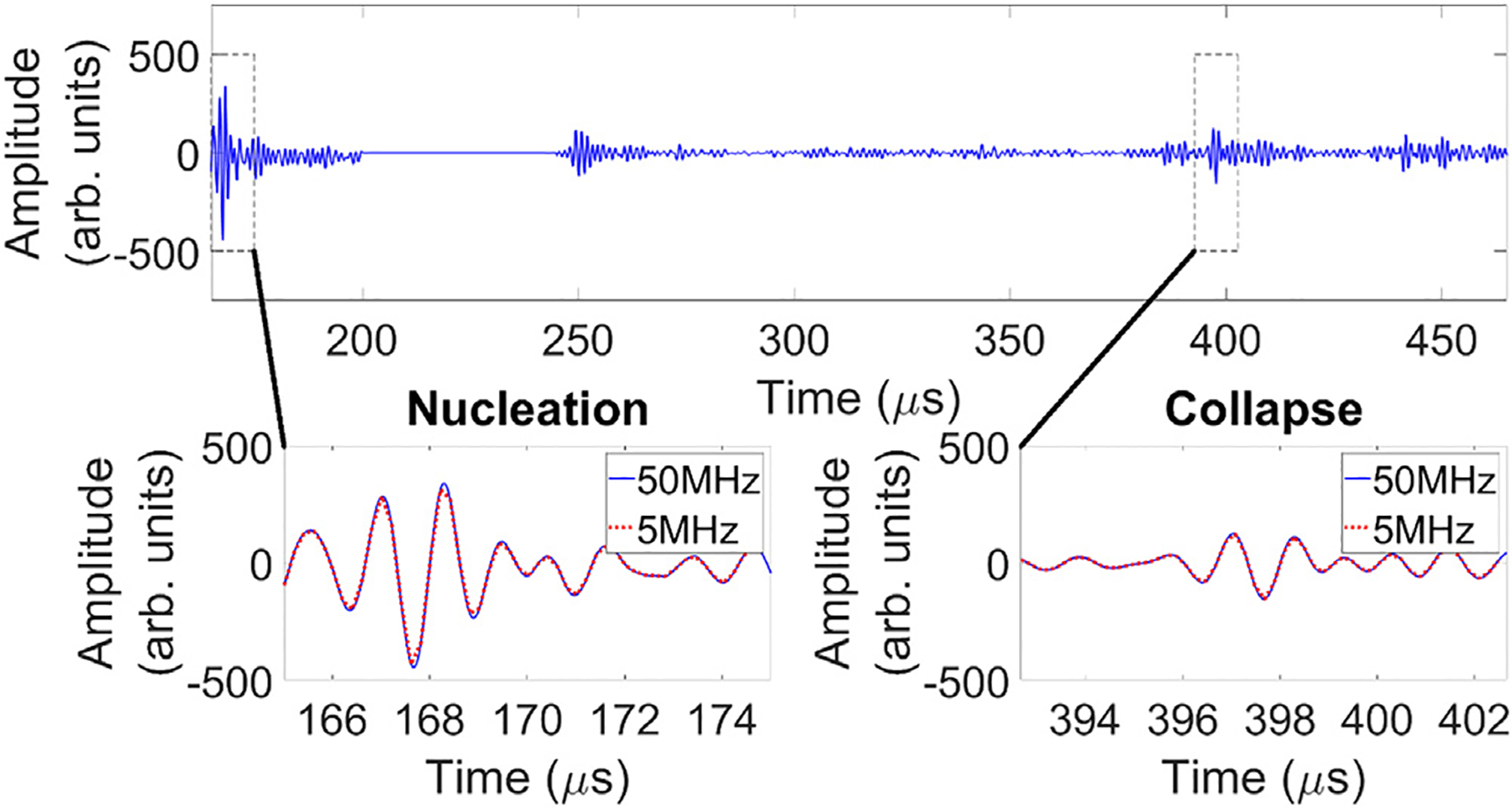
Example ACE Waveform. The sub-figures on the bottom row are zoomed windows of the nucleation and collapse shockwaves as measured by one transducer element of the histotripsy array. Although the analog-to-digital converter (ADC) can sample up to 50MHz, data was captured at 5MHz during ablations to decrease the volume of the resulting data.

ACE-signals were captured using the receive capable elements (Rx) of the histotripsy arrays following every other pulse of treatment [37] (Figure 3). To further reduce the data volume, signals were acquired at a sampling frequency of 5 MHz. Receive data was sub-sampled in this way in order to manage the volume of recorded data while still maintaining the ability to sample bubble activity across the treatment volume. Phase-delays were applied to compensate for focal steering, then the timings and amplitudes of each shockwave within the acquired signals were calculated as follows and recorded. Given time *t* in 1:T, elements *e* in 1:E, and signal S(t,e):

(1) $$\text{Coherence}(t)=\frac{{\left(\sum_{1}^{E}S(t,e)\right)}^{2}}{\sum_{1}^{E}S{\left(t,e\right)}^{2}}$$

**Figure 4.**
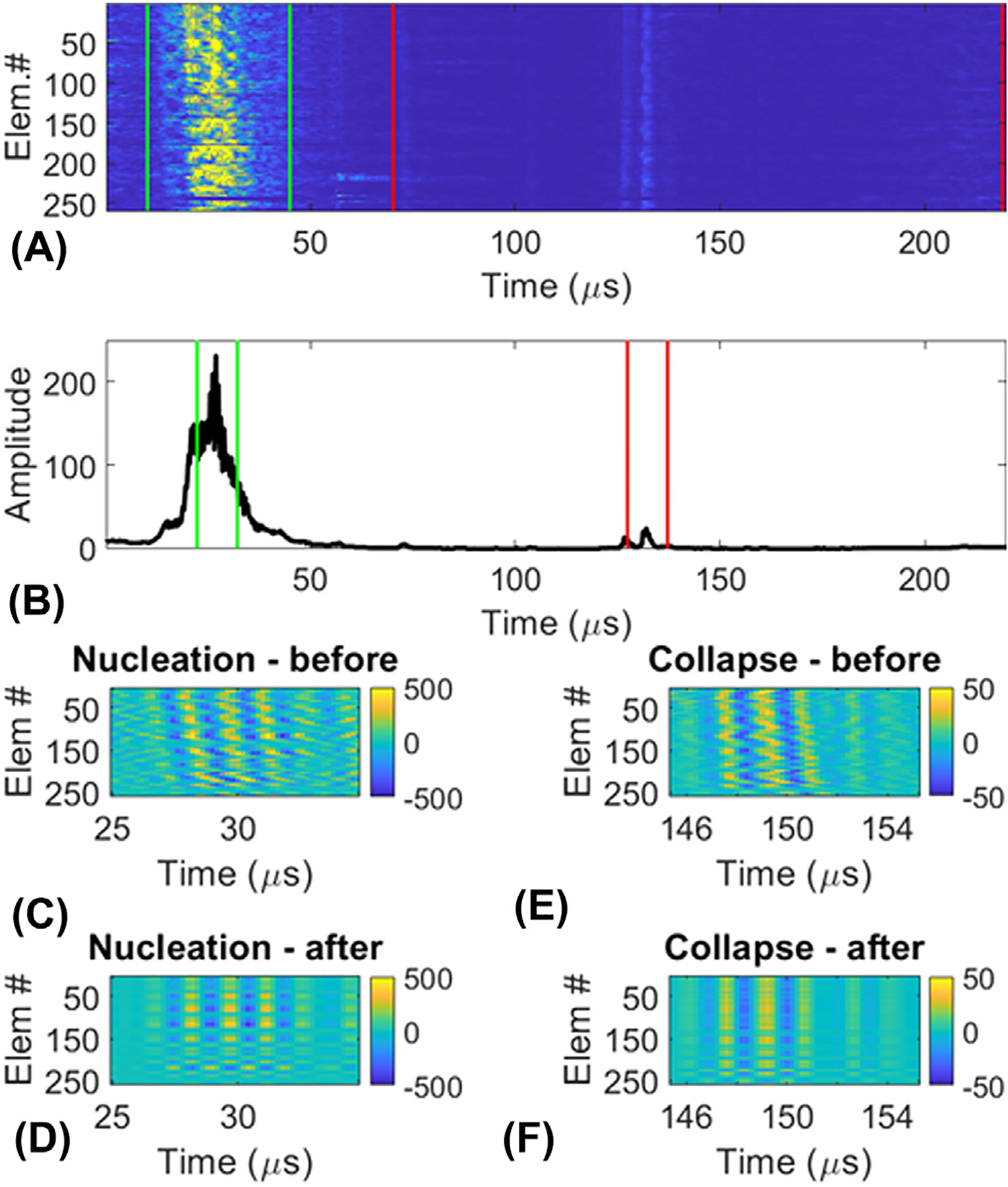
Shockwave measurement from an ACE signal. (A) Apply “rough” windows around the nucleation and collapse shockwaves in the time × numElements image, and calculate the coherence across both windows. (B) Peak-detect the nucleation and collapse shockwaves, and calculate “tight” (20 μs) windows around each one for an SVD filter. (C) Before and (D) after the SVD filter is applied to the “tight” nucleation window. (E) Before and (F) after the SVD filter is applied to the “tight” collapse window.

First, the coherence of the signal from a single histotripsy event measured across the elements of the acoustic array was calculated as described in Equation 1 following the metric introduced by Mallart and Fink [38] (Figure 4a). Because ACE-signals have high phase coherence compared to reflections from tissue, time windows for the nucleation and collapse signals could be determined via peak detection on the coherence [39,40] (Figure 4b). These 20μs windows were applied to the raw data, and singular value decompositions were calculated on the time-windowed data matrices containing nucleation or collapse signals from all elements [41] (Figure 4 c,d,e,f). All but the first eigenvalues were discarded to remove background noise and isolate the shockwaves [39,42,43]. Peaks in the SVD-filtered signals were detected to identify the shockwave timings and amplitudes for each focal-location.

Bubble lifespan is calculated as the mean difference between the nucleation and collapse shockwave arrival times measured across all focal locations. Then, bubble lifespan-slope was calculated as a series of linear-fits on the bubble lifespan as a function of ppl using a moving window (size 4 ppl). Finally, percent-reduction (i.e. normalized reduction in lifespan-slope) is calculated with Equation 2. Given pulse *p* in 1:P, and LifespanSlope(p):

(2) $$\%\text{Reduction}(p)=100*\left(1-\frac{\text{LifespanSlope}(p)}{\text{LifespanSlope}(1)}\right)$$

### Histology

Following treatment, each tissue block (embedded in agarose gel) was marked to indicate the positive X/Y/Z axes (relative to the histotripsy array elements’ coordinates) and was stored in 10% formalin for 72 hours to fix before storage in 70% ethanol. To prepare for histology processing, each tissue block was bisected using the ‘registration’ lesion to localize the damaged volume. After bisecting the samples, two notches were cut into the tissue outside of the treatment zone to preserve indicators of the orientation of the sample during treatment following histological processing as needed for co-registration with localized, ACE-signal assessments later. One diagonal notch was placed in the corner of the cube to designate the +X and +Z directions, and another triangular notch was placed midway along the face of the cube to designate the −Z direction (Figure 4). These notches allowed unique identification of the orientation of the sample within the histotripsy array during treatment. Finally, tissue samples were loaded in histology cassettes, embedded in paraffin, and stained using hematoxylin and eosin (H&E) to visualize their structures and the mechanical disruption generated within.

**Figure 5.**
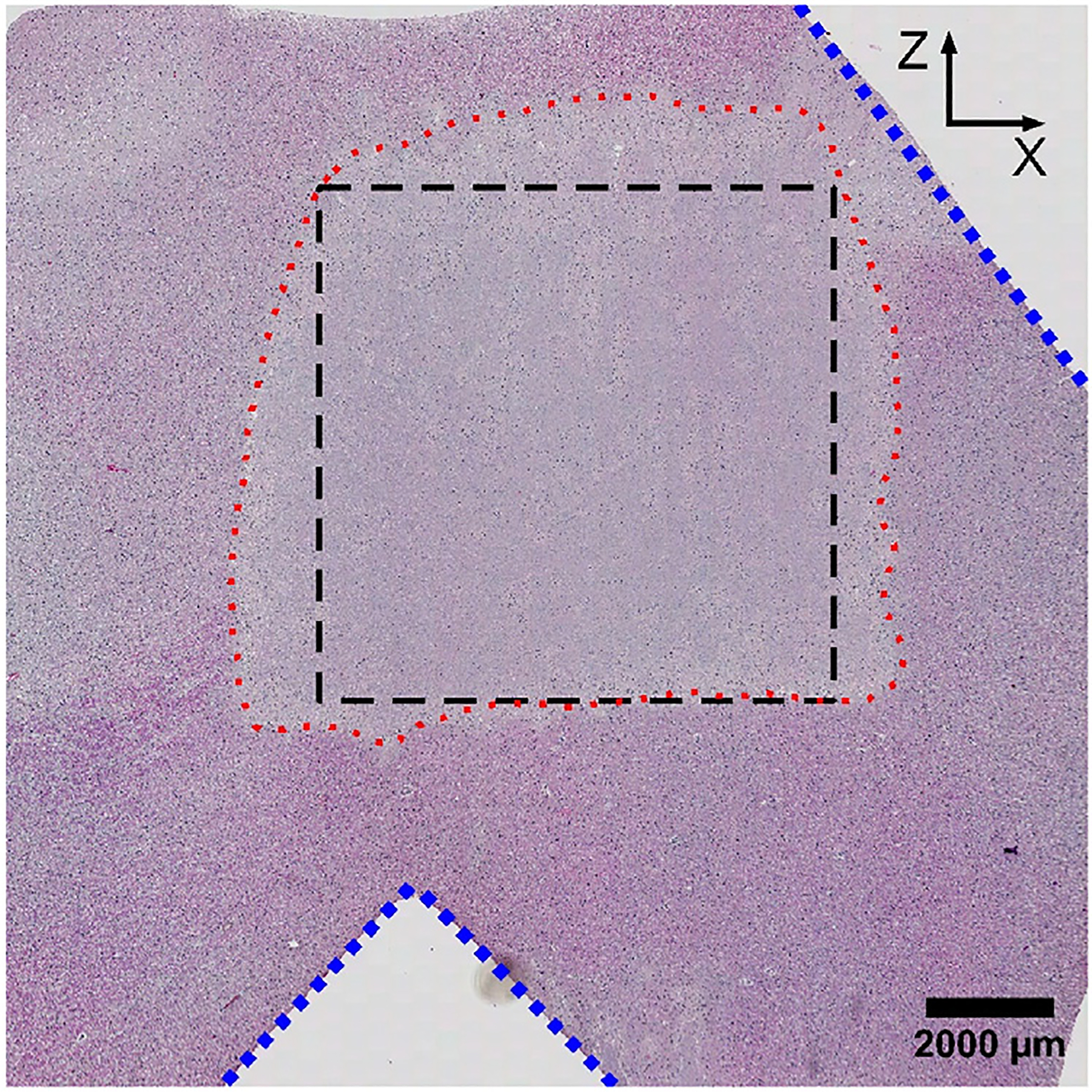
Sample H&E of treated bovine brain. Histotripsy was applied in the −Z -to- +Z direction. Blue dotted lines indicate cuts to register H&E and histotripsy array coordinate systems. The black dashed line indicates the prescribed treatment area. The red dotted line indicates ablated tissue.

Following staining, ground-truth damage results were assessed by a board-certified surgical pathologist (Jiaqi Shi). Damaged regions, similar to the region outlined in black in Figure 5, were manually segmented on each slide for analysis. Then the pathologist, blinded to the treatment applied to the tissue, graded the damage in each region based on the overall percentage of tissue necrosis and the remaining areas of residual structures (e.g., epithelial cells, neurons, blood vessels, stroma, glia, etc.) using the following grading scales [44]:

Necrosis score: 0 (0%), 1 (1–25%), 2 (26–50%), 3 (51–75%), 4 (76–95%), 5 (>95%)

Residual Structure: 0 (none present), 1 (patchy/small areas), 2 (patchy/large areas), 3 (diffuse areas), 4 (intact). Several samples were omitted from the final analysis due to the histology slices not intersecting the center of the ablation lesion. This resulted when larger tissue samples surpassed the ‘registration’ lesion, and clean slices of the center of the ablation zone could not be procured following histotripsy exposure. The following samples were removed from their respective tissue-type/acoustic-dose groups: 2 brain-20ppl, 1 liver-3ppl, 1 liver-5ppl, 1 kidney-5ppl, 1 kidney-10ppl.

### ACE Correlation with Pathology

As the pathologist evaluations are discrete measurements, the Spearman correlation was used to evaluate the efficacy of using ACE-signals to predict tissue disruption. The lifespan, lifespan-slope, and percent-reduction from the last pulse of each ablation were correlated against the corresponding necrosis scores and residual structure scores.

In addition, ideal thresholds for binary evaluations of no/partial damage vs. near-complete/complete damage were determined for each of the three ACE-features (lifespan, lifespan-slope, percent reduction). For the ground-truth data, a necrosis grade 4 and 5 was used as the threshold for near-complete/complete damage. Although grade 4 retained some viable tissue, most of the residual tissues are detached, minute islands of cells suspended in necrotic debris with no blood supplies and would likely not survive. For the experimental data, final lifespan/lifespan-slope/percent-reduction was used to predict the presence of near-complete/complete damage. ROC curves were calculated by sweeping threshold values for each of the ACE-features, and calculating the corresponding sensitivities (Eq. 3) and specificities (Eq. 4) to the pathological results. Area-under-the-curve (AUC) was calculated using integration with the trapezoidal method. The ideal threshold for each the ACE-feature/tissue-type pair was identified as the value with the greatest F1-score (Eq. 5). For this value, the “peak” sensitivity, specificity, accuracy (Eq. 6), positive-predictive value (PPV) (Eq. 7), and negative-predictive value (NPV) (Eq. 8) are calculated. Without a defined clinical standard for damage monitoring of mechanical ablation, validation of this technique was performed by comparing its efficacy against those reported for other methods [7,14,17,20].

For true-positive counts *TP*, true-negative counts *TN*, false-positive counts *FP*, and false-negative counts *FP*:

(3) $$\text{Sensitivity}=\frac{\text{TP}}{\text{TP}+\text{FN}}$$

(4) $$\text{Specificity}=\frac{\text{TN}}{\text{TN}+\text{FP}}$$

(5) $$F_{1}=\frac{2\text{TP}}{2\text{TP}+\text{FN}+\text{FP}}$$

(6) $$\text{PPV}=\frac{\text{TP}}{\text{TP}+\text{FP}}$$

(7) $$\text{NPV}=\frac{\text{TN}}{\text{TN}+\text{FN}}$$

(8) $$\text{Accuracy}=\frac{\text{TP}+\text{TN}}{\text{TP}+\text{TN}+\text{FP}+\text{FN}}$$

## Results

### Pathological Analysis

**Figure 6.**
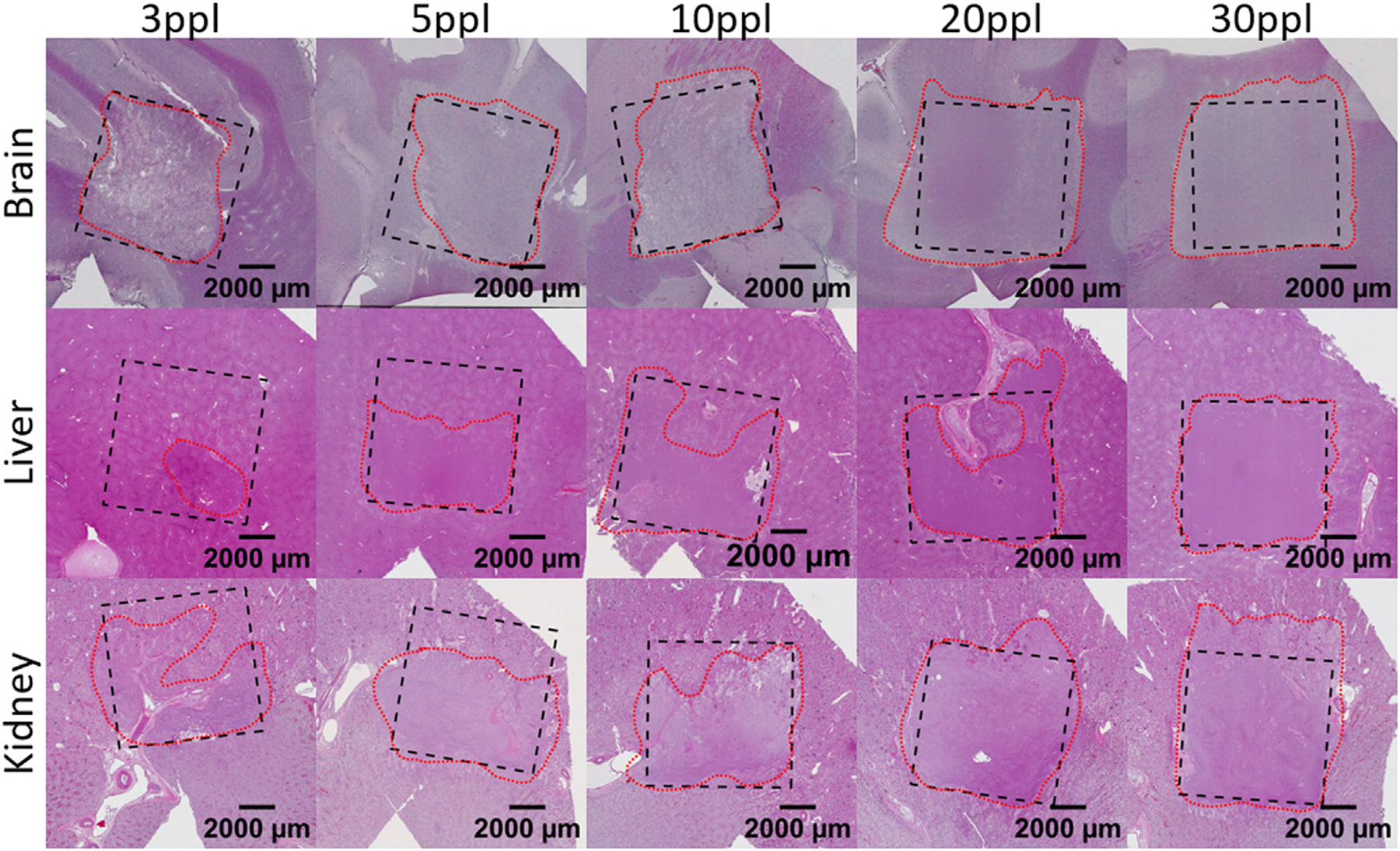
Sample full slide scans of H&E staining for each tissue type and exposure count. The black, dashed line indicates the prescribed treatment area. The red, dotted line indicates ablated tissue.

**Figure 7.**
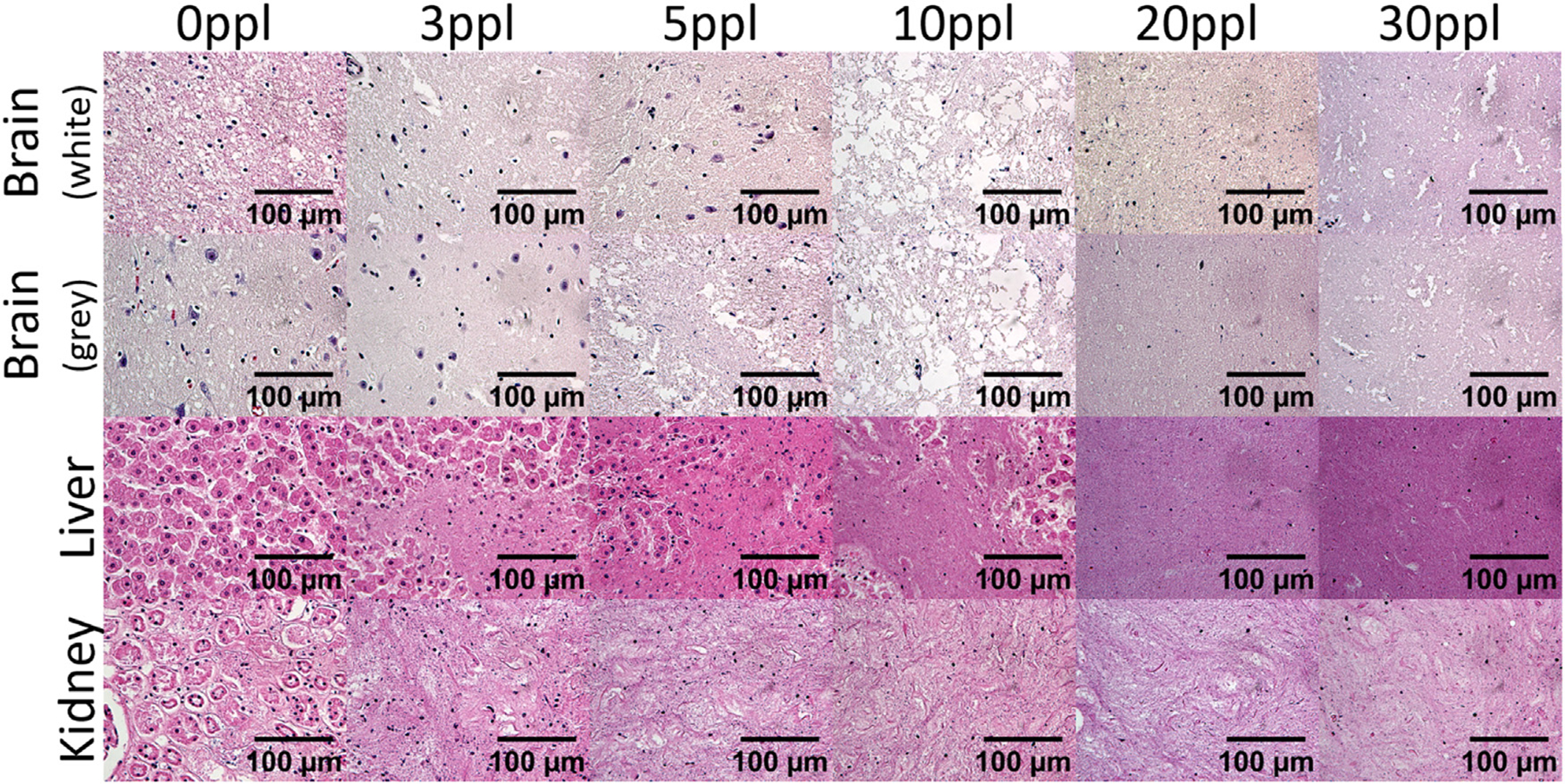
Sample high magnification (400x magnified) images of H&E staining for each tissue type and exposure count.

The level of cellular disruption was observed to increase across the treated volumes with increasing ppl in all tissue types (Figure 6,7). At lower acoustic doses (3–5 ppl), damage appeared to result in the formation of ‘pores’ in the tissue, with isolated pockets of cellular lysis. At increased exposure levels (10 ppl), these pores began expanding into one another and merging, and a significantly higher degree of cell lysis is noted. Further ablation resulted in nearly homogenous cell lysis by 20 ppl in both the liver and kidney tissues, and by 30 ppl in the brain tissue (Figure 7). Some sub-regions, particularly areas near an inhomogeneity in the tissue, were observed to still contain isolated pockets of cells free-floating in the necrotic homogenate.

The presence of heterogeneities in the tissue samples, such as vasculature, stroma, portal tracts, or other tissue interfaces, were observed to significantly impact the resulting damage outcomes (e.g. Figure 6 Liver 20ppl). In the low-ppl treatments of brain tissues for example, it was observed that white matter was damaged more quickly than grey matter (i.e. progressed through poration/lysis/homogenization in fewer ppl) (Figure 6 Brain 3/5/10ppl). High-ppl brain samples resulted in total homogenization across the treatment volume for both white and grey matter. As spatial segmentation between white and grey matter could not be effectively determined on the slides post-treatment, ACE-signal and pathologist measurements described here treat them as the same. However, the difference in damage behavior noted between the two tissue subtypes likely affects the final damage results.

**Figure 8.**
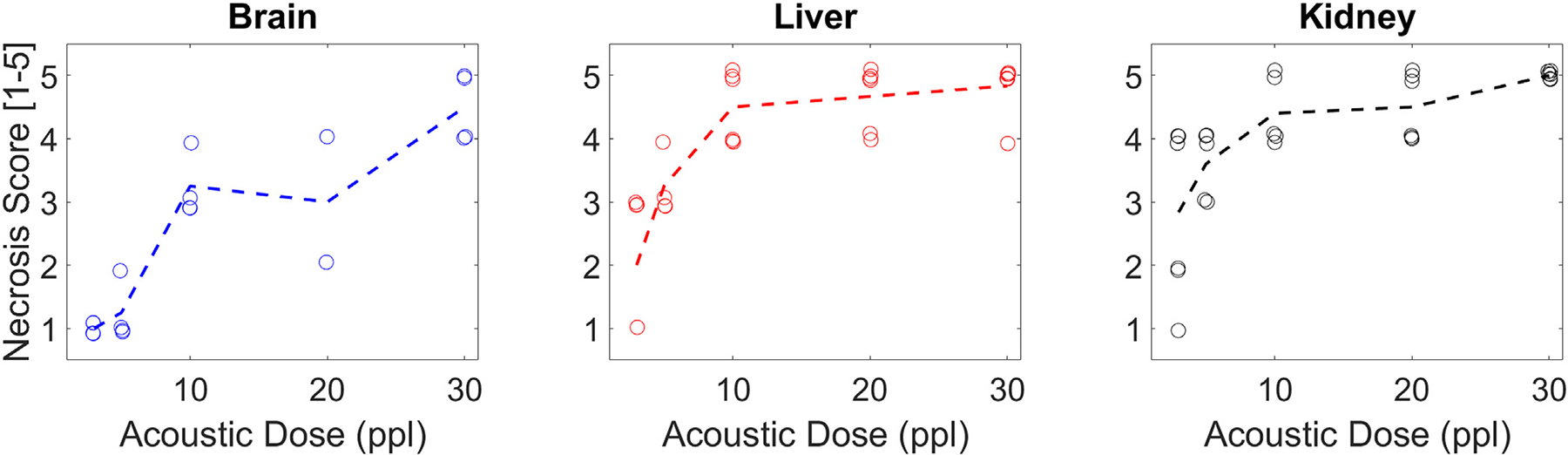
Necrosis score vs. ppl. Dotted line indicates the mean across samples by ppl. Necrosis score is a discrete metric, so some jitter has been added to visualize overlapping points.

**Figure 9.**
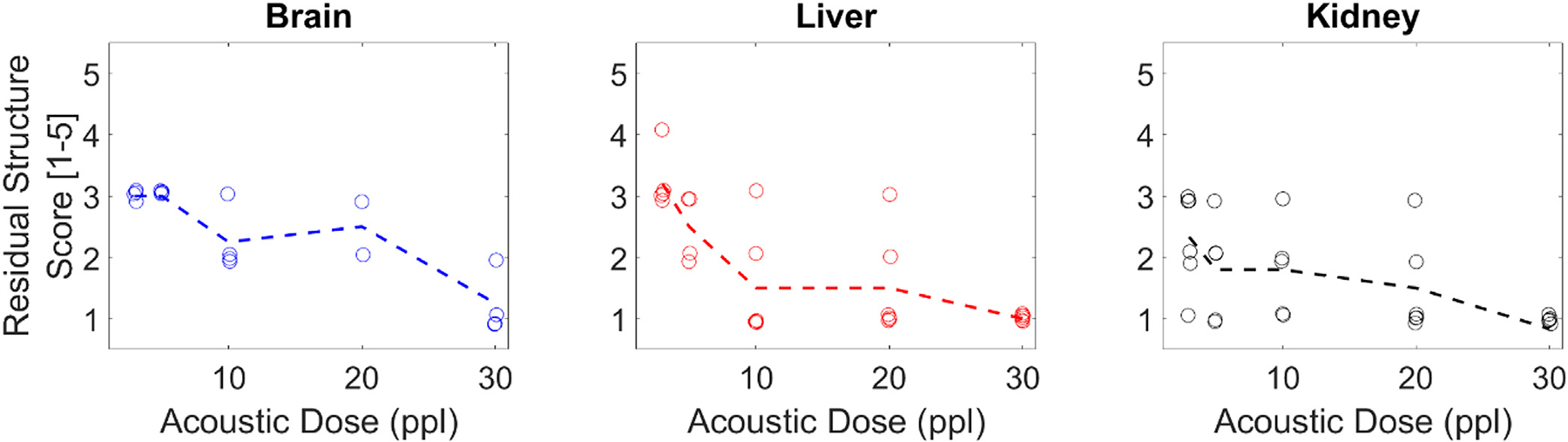
Residual structure score vs. ppl. Dotted line indicates the mean across samples by ppl. Residual structure score is a discrete metric, so some jitter has been added to visualize overlapping points.

Pathological quantifications of the H&E slides indicated increasing levels of damage with increasing ppl in all tissue types (Figure 8 & 9). The necrosis scores increased fastest during initial exposures and plateaued for higher exposure counts. The residual structure scores also decreased fastest during initial exposures for the liver and kidney, tests, but did not do so for the brain samples. Across the brain samples, the degree of damage stayed mostly consistent for each dose. In the liver and kidney tissues, samples treated using lower exposure counts (3–5 ppl) varied more in necrosis scores than the samples treated using higher ppl. No such significant variations as a function of dose were noted for the residual structure scores.

### ACE Analysis

**Figure 10.**
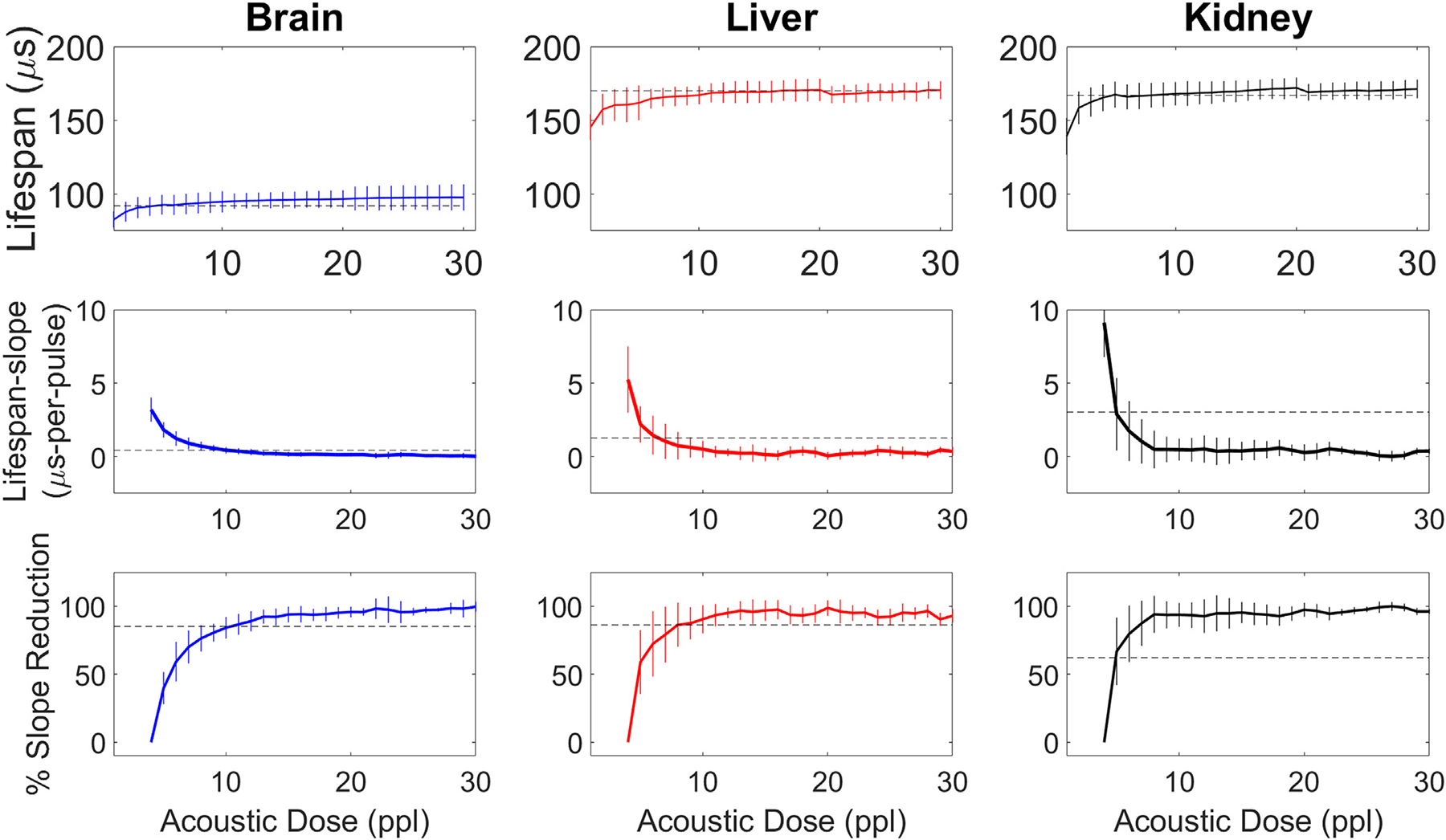
Mean bubble lifespan, lifespan-slope, and percent-reduction vs. ppl. Data is across all test samples & focal locations. Vertical lines indicate ±1 standard deviation. Horizontal, dashed lines indicate the binary-thresholds determined from Figure 12.

Measured from the ACE-signals, bubble lifespan increased with increasing acoustic dose for all treated tissue types. Bubble lifespans increased most rapidly during initial exposures before plateauing during later exposures. Relative changes in bubble lifespan in the brain tissues increased the least with increasing exposure level (20μs), followed by liver and kidney (50μs) (Figure 10). For reference, the inherent pulse-to-pulse variability in lifespans for bubbles generated in free field using the nucleation conditions described for these experiments was on the order of 5μs.

As expected, based on the asymptotic behavior of the bubble lifespans, the per-pulse changes in bubble lifespan, or lifespan-slope (Figure 10), were observed to decrease and approach 0μs/pulse as a function of increasing acoustic dose. Initial bubble lifespan-slopes were smallest in brain, then liver, then kidney. In addition, the standard deviation of the measured lifespan-slopes decreased with increasing dose. The standard deviation of lifespan slope started-to-finished at 1.62-to-0.23 μs/ppl in brain, 4.54-to-0.54 μs/ppl in liver, and 4.62-to-0.31 μs/ppl in kidney. Standard deviation trends were similar for percent-reduction. While percent-reduction data from all tissue types was bounded from 0 to 100 percent, bubbles generated in kidney tissue approached 100% reduction the fastest (i.e. approached the asymptote in the fewest ppl), while bubbles generated in brain tissue approached 100% reduction slowest (Figure 10).

### ACE correlation with Pathology

**Figure 11.**
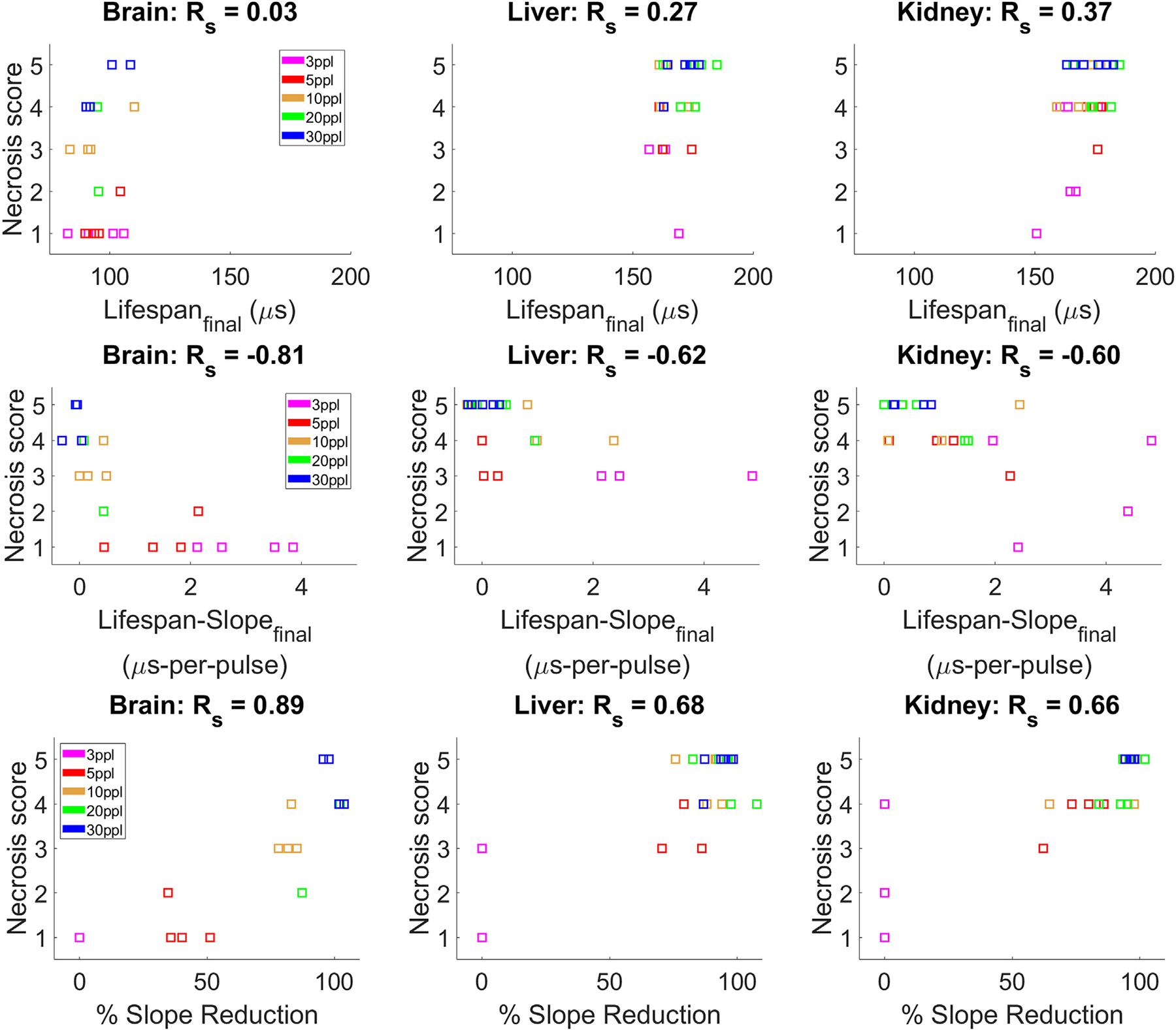
Final lifespan, lifespan-slope, and percent-reduction correlated with necrosis score. Each point represents the result of a single sample, and colors indicate the corresponding exposure count.

Lifespan correlated poorly with the assessed necrosis score, while Lifespan-slope correlated well with the assessed necrosis score; the calculated Spearman coefficients were observed to be 0.81, 0.62, and 0.60 in brain, liver, and kidney respectively (Figure 11). Percent reduction also correlated well with Spearmen coefficients of 0.89, 0.68, and 0.66 in brain, liver, and kidney respectively. Notably, there is greater variation in lower-dose acoustic samples as compared to higher-dose samples. Residual structure scores correlated poorly with all ACE-features (Spearman coefficients ≤ 0.15). The one exception was between residual structure score and percent reduction in brain tissue, which was observed to have a Spearman coefficient of 0.51.

**Figure 12.**
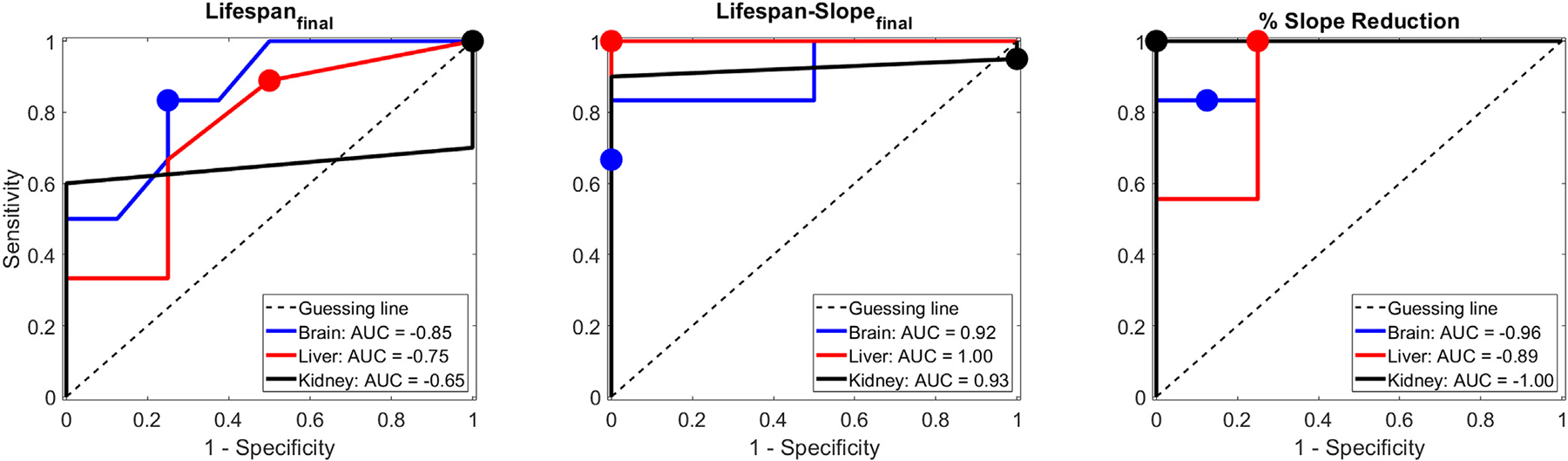
ROC curves comparing all tissue-types against A) Lifespan, B) Lifespan-slope, and C) Percent-reduction.

To determine a binary evaluation of whether the tissue damage was near-complete/complete (i.e. necrosis score equal to or greater than 4), ROC-curves comparing final bubble lifespan, lifespan-slope, and percent-reduction to necrosis score and the AUC were calculated for every ACE-feature/tissue-type pair (Figure 12). The ideal threshold for each ACE-feature was determined as the value with the greatest F1-score (Table 1). AUC values of predictions from lifespan were lower than the AUC values measured from lifespan-slope and percent reduction. When comparing ACE-features, AUC generally increases from lifespan to lifespan-slope to percent reduction for each tissue type (the exception being liver: lifespan-slope to percent reduction). Lifespan-slope and percent reduction shared similar accuracies, PPV’s, and NPV’s across all tested tissue types, while lifespan was slightly lower in all metrics.

## Discussion

In this study, it was observed that bubble lifespan as derived from ACE-signals acquired during histotripsy treatments of bovine brain, liver, and kidney tissue, increased and plateaued with the cumulative level of acoustic dose. Similarly, pathological evaluations of necrosis outcomes in the treated tissues expressed a trend of increasing-and-plateauing necrosis grading with increasing cavitation exposure. Threshold values for final bubble lifespan, lifespan-slope, and percent reduction were determined for each tissue to evaluate near-complete/complete damage with high sensitivity and specificity in all tested tissue types. The match between the lifespan-slope and percent-reduction change assessed from the measured ACE-signals, and the necrosis outcomes in treated tissues suggest that this may provide an avenue for actively assessing/predicting necrosis outcomes, particularly near-complete/complete damage, across different tissues during histotripsy treatments; eliminating the need to prescribe worse-case scenario histotripsy-doses. The correlations between cavitation lifespans and residual structure scores are significantly weaker than those between necrosis scores. This is likely due to the previously reported enhanced resistance to cavitation induced damage in these structures and suggests alternative metrics are required if monitoring the degree of ablation of these structures is required during therapy.

Slope reduction was observed to be the most reliable ACE-feature for predicting near-complete/complete damage by histotripsy ablation. From lifespan to lifespan-slope to percent reduction, both the AUC and the Spearman-correlations increased, suggesting that the normalization steps in each transformation effectively removed noise from the previous measurement. All ACE features were observed to have high-sensitivity to histotripsy-induced damage, but lower specificity; this is likely due to the lower number of negative samples (i.e. no/incomplete damaged). Despite this, slope reduction was observed to have PPV and NPV greater than or equal to 0.9 across all tested tissue types. This suggests that ACE-signals, especially percent reduction, are an effective tool for predicting whether histotripsy has achieved near-complete/complete tissue disruption.

ACE-signal measurements of bubble lifespan performed similarly as compared to other studied histotripsy-monitoring modalities (Table 2). Bader et al have demonstrated methods using MRI (T1,T2,ADC), plane-wave ultrasound, and passive-cavitation imaging (PCI) to predict tissue liquefaction in red-blood cell phantoms, prostate phantoms, and ex-vivo liver [7,14]. Other studies have demonstrated the use of color doppler and shear-wave elastography (SWE)[17,20] for monitoring histotripsy induced damage in phantoms, liver and kidney. Compared to the current clinical practice, ultrasound imaging, cavitation lifespan performed equivalently, while lifespan-slope and percent reduction demonstrated enhanced ability to detect histotripsy induced damage. ACE-signal based assessments performed similarly to PCI, and were an improvement on the MRI methods. Correlation coefficients between histotripsy induced damage were smallest for cavitation lifespan and greatest for percent reduction; which approached but did not exceed those reported for these color-doppler and shear-wave elastography studies.

This study was motivated by the need for feedback methods for clinical histotripsy treatments, with two possible control loops in mind: One potential implementation would be to monitor the evolution of the ACE-signal metrics in real time and terminate treatment once a determined threshold is achieved. One target of this study was to establish metrics and thresholds for this method of feedback loop. Another possible method would be to perform a minor pretreatment dosing-study to probe the ideal PPL on a patient/tissue/target-specific setting. In this case, a single-point (or a small subset of focal locations) would be exposed to the “worse-case scenario” histotripsy dose, and the ACE-signals would be recorded. Then, signals would be analyzed to determine the PPL necessary to achieve a determined threshold of damage based on the measured the ACE-features. A further set of experiments implementing either of these methods to generate lesions with pre-determined levels of tissue disruption would be necessary to validate them.

While the measured bubble lifespan-slopes correlated reasonably well with necrosis outcomes in the treated tissues, significant sample-to-sample variabilities in both pathologist and ACE-feature quantifications were noted throughout the experiments. Several factors likely contributed to these outcomes, but heterogeneities (e.g., blood vessels, portal tracts, stroma, etc.) within the treated tissues were perhaps the most significant, and the problems they introduced were several-fold. While reasonable efforts were made when sectioning the tissue samples prior to treatment to avoid obvious heterogeneous inclusions such as large vasculature, the parenchyma of the treated tissues were themselves heterogeneous, and necrosis outcomes varied substantially in space when the treatment volume spanned different parenchymal components (e.g., grey vs white matter in the brain, or renal medulla vs cortex in the kidney). As necrosis was scored based on the percentage of necrosis across the entire treatment region, the reported scores do not capture any details about the spatially localized variabilities in damage outcomes. Correlations between the bubble lifespan metrics and necrosis outcomes were likewise made based on the spatially averaged values of the lifespan metrics, which likely had a negative impact on the correlational outcomes. As each ablation region measured approximately 1cm^2^, it was not feasible to manually segment and provide spatially mapped necrosis scores for all the samples treated in this study. This could be a subject for future study where variations in cavitation susceptibility would be examined on a subregional basis.

It is worth noting that in all tissues, the bubble lifespans typically began to plateau at acoustic doses of ~5–10 ppl and roughly when the necrosis scores began reaching or exceeding 4. As a result of the fixed exposure count design of the experiments, there were relatively few ablations with low necrosis score in the treated tissue samples. This outcome may have been a consequence, in part, of the small spacing between adjacent steering locations in the treatment volume (0.5 mm), which would have led to significant overlap between cavitation (Ø 1.5 mm) generated at adjacent treatment locations and higher effective exposure counts at each steering location than the stated ppl’s indicate. Nonetheless, although the skew in necrosis score outcomes likely had a negative impact on our ability to *quantitatively* correlate them with the bubble lifespan data across a wide range of necrosis scores, particularly at lower damage levels, the bubble lifespan still enabled accurate identification of whether or not a given treatment had reached a necrosis score ≥4. Although being able to quantitatively track treatment progress is desirable, for instance to assess whether damage in certain regions is progressing slower than in others and increase treatment there, or to hit specified damage levels to optimize potential immune responses [25,26], the binary ability to assess whether damage is near-complete/complete is still highly valuable for ensuring efficient, repeatable treatments. Such feedback can ensure the most efficient treatment across different tissue types without necessitating the use of worst-case scenario histotripsy doses.

**Figure 13.**
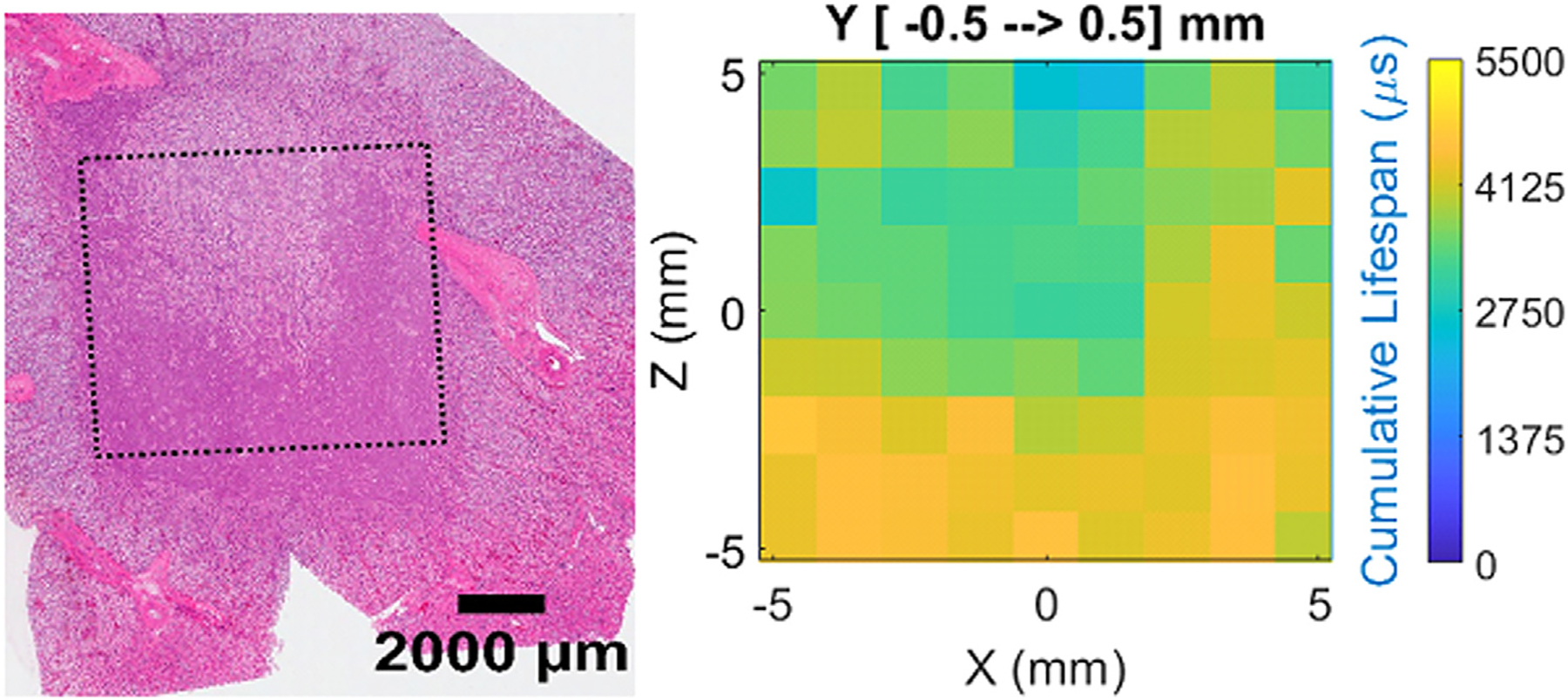
(A) H&E scan showing heterogeneous tissue damage. (B) ACE lifespan heatmap generated from signals acquired during the corresponding ablation.

While the treatment parameters of this ablation study were not optimized to produce fine grained spatial maps of necrosis outcomes in treated tissues, the spatially mapped bubble lifespans did accurately reflect the qualitative damage outcomes observed macroscopically (Figure 13). In particular, the cumulative bubble lifespan (Equation 9) assessed at each focal location and across all delivered pulses, agreed well with morphological observations of damage outcomes in the treated tissues. In regions where the cumulative bubble lifespan was lower, less damage was observed than in regions where the cumulative bubble lifespan was highest. For a cartesian volume *x,y,z* in [1:X,1:Y,1:Z], PPL *p* in 1:P, and Lifespan(x,y,z,p):

(9) $$\text{CumulativeLifespan}(x,y,z)=\sum_{p=1}^{P}\text{Lifespan}(x,y,z,p)$$

The histotripsy transducer used to treat the brain samples (transcranial array) was different from that used to treat the liver and kidney samples (abdominal array). The use of the different histotripsy transducers in this study was predicated on the notion that the different types of tissues should be treated using transducers with a clinically relevant geometries (based on existing clinical histotripsy and HIFU systems for the relevant applications). However, although the focal pressures of both arrays were set in order to generate equivalently sized bubbles, the magnitudes of the bubble lifespans, as well as the overall increases in lifespan as a function of acoustic dose, varied significantly and in an unexpected way between the tissues treated using the different arrays. Namely, the bubble lifespans, and changes thereof, in treated brain tissues were smaller than those in liver and kidney tissues, despite brain tissue being the softest of the three [22,23] and being treated using a higher focal pressure (40 MPa) than the other two (30 MPa). These outcomes are likely a result of the shapes of the bubble clouds generated by the respective arrays, where the aspect ratios (axial diameter / transverse diameter) of bubbles generated using the transcranial array (as measured in the free field) were typically <1.25, while those generated using the abdominal array approached aspect ratios closer to 2. The shapes of cavitation bubbles are known to have a significant impact on their dynamics, which may have contributed to the observed differences in the magnitudes of the bubble lifespans in the tissues treated using the different arrays [45–47].

## Conclusion

In this study, bubble lifespan calculated from ACE-signals measured during large-volume histotripsy ablations expressed the same increasing and plateauing trends with increasing acoustic dose in bovine brain, liver, and kidney tissues. Progressively increasing levels of damage were also observed with increasing cavitation exposure, which were evaluated with histological necrosis and residual structure gradings post-treatment. Final bubble lifespan slope and precent-reduction predicted damage outcomes beyond necrosis grade 4 with high sensitivity and specificity in all tissue types. Several sources of variation were identified which negatively impacted the efficacy of the quantitative ACE-feature and histological damage measurements. Nevertheless, correlations between lifespan-slope measured from ACE-signals and histological scores indicate that it may be used to actively predict near-complete/complete histotripsy ablation during treatments.

## Figures and Tables

**Table 1:** Binary ACE Evaluation of Histotripsy-induced Tissue Homogenization

ACE-feature	Tissue	AUC	ACE-threshold (μs)	Sensitivity	Specificity	Accuracy	PPV	NPV
Lifespan_final_	Brain	**0.85**	91	0.83	0.75	0.79	0.71	0.86
	Liver	**0.75**	157	0.89	0.50	0.77	0.80	0.67
	Kidney	**0.65**	150	1.00	0.00	0.95	0.95	-
Lifespan-Slope_final_	Brain	**0.92**	0.19	0.83	1.00	0.93	1.00	0.89
	Liver	**1.00**	1.20	1.00	1.00	1.00	1.00	1.00
	Kidney	**0.93**	3.39	1.00	0.00	0.95	0.95	-
% Slope Reduction	Brain	**0.96**	87.30	0.83	1.00	0.93	1.00	0.89
	Liver	**0.89**	70.60	1.00	0.75	0.92	0.90	1.00
	Kidney	**1.00**	62.60	1.00	1.00	1.00	1.00	1.00

**Table 2:** Performance of histotripsy monitoring techniques in-vitro

Monitoring Technique		Tissues	Sensitivity	Specificity	Correlation Coef.
Plane-wave imaging		RBC phantom	0.88	0.97	-
		Prostate phantom	0.49	0.95	-
		Liver	0.85	0.71	-
		Kidney	-	-	0.99
Color Doppler		Liver	-	-	0.95
Shear wave elastography		Kidney	-	-	0.91
Passive cavitation imaging		Prostate phantom	0.81	0.93	-
		Liver	0.86	0.88	-
MRI	T_1_	RBC phantom	0.50	0.87	-
		Liver	0.34	0.89	-
	T_2_	RBC phantom	0.30	0.95	-
		Liver	0.81	0.80	-
	ADC	RBC Phantom	0.00	1.00	-
		Liver	0.84	0.83	-
ACE	Lifespan	Liver	0.89	0.50	0.27
		Kidney	1.00	0.00	0.37
		Brain	0.83	0.75	0.03
	Lifespan-slope	Liver	1.00	1.00	0.62
		Kidney	1.00	0.00	0.60
		Brain	0.83	1.00	0.81
	Slope-reduction	Liver	1.00	0.75	0.68
		Kidney	1.00	1.00	0.66
		Brain	0.83	1.00	0.89

## Data Availability

Contact the corresponding author for access
